# Audit of surgical services in a teaching hospital in Addis Ababa, Ethiopia

**DOI:** 10.1186/s13104-019-4709-y

**Published:** 2019-10-22

**Authors:** Hailu Wondimu Gebresellassie, Girmaye Tamerat

**Affiliations:** 0000 0001 1250 5688 grid.7123.7 CHS, SOM, AAU, Addis Ababa, Ethiopia

**Keywords:** Audit, Zewditu, Emergency, Elective, Mortality, Operation

## Abstract

**Objective:**

The aim of the study is descriptively analyze services in terms of surgical care, performance and outcome in department of surgery of ZMH which is a university affiliated general hospital in Addis Ababa, Ethiopia. Data on mode of admission, procedures done and outcome were collected from a monthly audit report, patients file. Information on the number of operating days missed and patients cancelled after being scheduled for surgery were collected from OR logo book.

**Result:**

Emergency operations constitute 57.4% of all operations. Appendectomy is the commonest emergency operation accounting for 41.5%. Thyroid and gallbladder surgeries were the most common elective operations accounting for 23.5% and 22% respectively. There were 26 and 2 deaths from the emergency and elective operations making the postoperative mortality rate of 2.8% and 0.02% respectively. The average hospital stay of a patient is 3.74 days. 23 of 211 (14.7%) operation dates were missed for various reasons. 81 of 693 (11.7%) elective operations were not done the first time they were scheduled. In conclusion this study showed emergency surgery out number elective surgeries, unacceptably high number of operation days are missed and scheduled surgeries are cancelled.

## Introduction

Audit in a clinical setting is the collection of data for the purpose of setting professional standards, assessing clinical performances and modifying the clinical practice [1], unlike its usual association with accounting which implies the numerical review by an outside investigator for the prevention of fraud.

In recent years studies had shown the importance of surgery in human health and welfare. Among the 51 million people who died in 2012, 17 million suffered from a disease that needed surgical service [2].

A recent study on the global burden of postoperative death showed that postoperative deaths account for 7.7% of all deaths globally and half of these occur in LMICs [3].

Emergency surgery represents over 50% of general surgical practice in UK. This rises in some hospitals, which provide regional Accident and Emergency services, to nearly 70%, and acute abdominal pain represents approximately half of all emergency surgical admissions [4]. Non traumatic acute abdomen represented 54% of general surgical admissions in Saudi Arabia [5].

In Ethiopia, a structured program for the clinical audit is not available. It is not a regular practice to conduct surgical audit routinely therefore proper clinical data is not available which can be reviewed and analyzed in terms of morbidity, mortality and other clinical outcomes in order to improve the overall clinical practice.

In this study due the lack of clinical data on surgical audit especially surgical emergency audit, where no comparative figures were available from different institutions, we had no option than to rely on the limited data available elsewhere for comparison.

## Main text

### Materials and methods

ZMH is university affiliated hospital in the center of city Addis Ababa, Ethiopia with over 120 beds (42 adult and 6 pediatric surgical beds). It has four the four major departments (internal medicine, Obstetrics/gynecology, Pediatrics).

Data on diagnosis, mode of admission, procedures done (if any) and outcome of patients seen and managed in the department of surgery from June 1, 2016 to May 30, 2017 were retrieved and descriptively analyzed.

The source of the data were patients file, outpatient registration book, inpatient registration book, operation theatre registration books and the monthly audit report of department.

#### Study design

This is a retrospective descriptive analysis of surgical services and care at department of surgery. Ethical approval and clearance were gained from AAU-MF IRB office.

### Result

4629 emergency and 4892 new non-emergency patients were seen in department i.e. on the average 12.7 patients per day. 2311 of 4629 (49.9%) were patients with minor conditions which does not need admission or observation (Tables 1, 2). There were 1640 admissions to surgical ward, 866 (52.8%) of which were emergencies. 144 of 1640 (8.8%) admissions were managed conservatively (or surgery deferred for various reasons). There were 240 emergency consultation almost all from department of pediatrics.

**Table 1:** Emergency and elective surgeries department of surgery, Zewditu Memorial Hospital a Teaching Hospitals Experience in Addis Ababa, Ethiopia

Procedure or diagnosis	Frequency	Percentage	Elective procedures	Frequency	Percentage
Appendectomy	388	41.5	Thyroid surgeries	163	23.5
Generalized peritonitis 2nd to appendicitis	48	5.1	Cholecystectomy	152	21.9
LBO	16	1.7	Prostatectomy	88	12.7
SBO	24	2.6	Hernia repair	75	10.8
Perforated PUD	26	2.9	Perianal procedures	53	7.6
Blunt abdominal trauma	16	1.7	mastectomies	34	4.9
Penetrating abdominal trauma	18	1.9	Colostomy closure	19	2.7
Re-laparotomy	16	1.7	CBD exploration	8	1.2
Various abscess drainage	127	13.6	Vagotomy + GastroJejenostomy	9	1.3
Debridement	18	1.9	Orchidopexy	13	1.9
Chest tube insertion	50	5.3	REEA for redundant sigmoid	29	4.2
Others	187	20	Others	50	7.2
Total	934	100	Total	693	100

**Table 2:** service, care, and outcome department of surgery, Zewditu Memorial Hospital a Teaching Hospitals Experience in Addis Ababa, Ethiopia

Operation theatre	Operations	Operation theatre activity	Number (%)
OR activity	Emergency operations	Emergency operations done	934 (57.4% of operations)
	Elective operations	Scheduled	774
		Operated	693 (89.5% of scheduled)
		Cancelled	81 (10.5% of scheduled)
		OR days missed	23 (10.9% of operation days)
		Total OR days	211
	Total number of patients operated	Emergency and elective	1627 (100%)
Surgical referral clinic	Type of patients	Number of new patients seen in surgical referral clinic	4892 (45.8% of patients seen in referral clinic)
		Number of Repeat/follow-up patients seen in surgical referral clinic	5802 (54.3%)
		Number of patients seen in surgical referral clinic	10,692 (100%)

There were 866 emergency and 774 elective admissions during the same period. 934 emergency and 693 elective major surgeries were performed. Emergency operations constitute 57.4% of all operations. Appendectomy is the commonest emergency operation accounting for 41.5% (Table 1). Thyroid and gallbladder surgeries were the most common elective operations accounting for 23.5% and 22% respectively (Tables 1, 2).

There were 26 deaths following emergency surgeries and 2 deaths following elective operations making the postoperative mortality rate of 2.8% and 0.02% respectively (Table 3). There were no intraoperative death. The average hospital stay of a patient is 3.74 days. 23 of 211 (14.7%) operation dates were canceled for various reasons. 81 of 774 (11.7%) scheduled elective operations were not done the first time they were scheduled (Table 3).

**Table 3:** Morbidity, mortality, average hospital stay department of surgery, Zewditu Memorial Hospital a Teaching Hospitals Experience in Addis Ababa, Ethiopia

Morbidity and mortality	Number per patients	%	Major post op cx	Number (%)	Cause of death	No [%]
Major morbidity (Grade 3-5 clavien-dindo complications)	56 of 1627	3.4	Deep surgical wound infection	20 (35.7)	Intractable septic shock	17 (65.4)
Non-operative death	3 of 1640 admissions	0.1	Postoperative abscess collection	18 (32.1)	Hypovolemic shock	2 (7.7)
Intra-operative death	0 of 1627	0				
Post-operative death after emergency surgery	26 of 934 operations	2.8	HAP	8 (14.3)	PTE	1 (3.9)
Post-operative death after elective surgery	2 of 693 operations	0.29	Anastomotic leaks	7 (11.9)	HAP	3 (11.5)
Average hospital stay	3.74 days		Hypo-calcemic tetany	2 (2.4)	Cardiac arrest	3 (11.5)
			Total	56 (100)	Total	26 (100)

### Discussion

This study showed that most of the patients (49.9%) presenting to surgical emergency departments like soft tissue injuries etc., do not deserve admission. This has also been the observation in Nigeria and Lahore, India, where 66.5% do not deserve admissions [6, 7]. Trauma related major neurosurgical (often head injuries) and orthopedic (such as long bone fracture) cases constitute 11.4%. The finding is almost similar to a tertiary hospital in Delhi where major orthopedic cases alone constitute 11.64% [8].

In low and middle income countries (LMICs), studies has showed that at least 60 percent of the surgical operations are performed for emergency patients [9]. Our study also showed 61% of all major operations performed were for emergency conditions. These fact suggests surgical emergencies may compromise day to day activities of operation theatres and calls for establishment of dedicated operation theatres and staff in emergency department.

The most common emergency surgery is simple or complicated appendicitis, accounting for 46.6%, 436 of 934 emergency procedures. A study done in this hospital from 1996 to 1998 showed that acute appendicitis accounts for 46.7% of admissions for acute abdomen [10]. These is in accordance with the study on pattern of surgical admissions to Tikur Anbessa where appendicitis was found to be the most common cause of acute abdomen and accounts for 37.4% of GI emergencies [11]. This has been confirmed by multicenter study involving high, middle and low income countries by global surgery collaborative group on mortality of emergency abdominal surgery in high-, middle and low-income countries [12].

An Observational Study of the Etiology, clinical presentation and outcomes associated with peritonitis in Lilongwe, Malawi also showed appendicitis to be the most common cause of peritonitis (22%) followed by gangrenous bowel obstruction and perforated peptic ulcer disease [13].This study and other studies in Ethiopia has showed perforated peptic ulcer disease to be 2nd most common cause of peritonitis and acute abdomen [14].

A study in Tikur Anbessa hospital has showed that that gallstone disease and thyroid surgeries are the most common indication for elective admission followed by BPH which is also the same finding in this study [11].

Surgical site infection including postoperative intra-abdominal collection is the most commonly encountered cause of morbidity, and mortality in this study, as in Rwanda, Kigali University Teaching Hospital [15]. SSI were found 12.3% patients in a study on SSI following gastrointestinal surgery across countries with different income level [16].

Septicemia, septic shock and multiple organ failure is what causes death in most postoperative surgical patients as seen in this study [10]. Mortality rate following emergency surgery is 2.8% (26 out of 934) while it is 0.29% following elective surgery and overall mortality rate 31 out of 1640 admissions (1.9%) in these study is much lower than reported in most literatures in Africa [6, 14, 17]. Perioperative mortality is considered a measure of quality of surgical care by WHO and a case study found that Perioperative mortality in Madagascar is between 2.5 and 3.3% while in Uganda it is 2.4% [18].

A Lancet report on global burden of postoperative death showed that 4.2 million people die within 30 days of surgery globally each year and half of this occurs in LMICs [3].

Our finding on overall mortality and mortality following emergency surgery are lower than that reported by a prospective multicenter study by globalsurg collaborative study of 1.9% and 6.8% respectively [3].

Cancellation of elective surgical operations is recognized as a major cause of emotional trauma to patients as well as their families. There is unacceptably high rate of cancellation of elective operations i.e. 10.5% although there are reports of higher rate in Africa as in a University Teaching Hospital in the Lake Zone, Tanzania [19]. Although this is a retrospective study, its findings describe the pattern and situation of general surgical practice in low income countries like ours.

### Conclusions

This study showed majority of the surgical patients are managed as an out-patient in the surgical emergency department and emergency surgeries account for most of the surgeries done in our hospital. The most commonly performed major emergency procedure is simple and complicated appendicitis. Significant number elective operations are cancelled for various reasons.

We suggest that audit should be carried out on regular basis in all hospitals since it provides insight and feedback to the hospital’s and surgeon’s performance. It serves as a tool to effectively improve the overall patient care in surgical emergencies [15].

## Limitation of the study

The limitation of this study is the fact that is a retrospective and relies on records/registries which are prone to be incomplete.

## Data Availability

There are no additional data and materials beyond those mentioned within the manuscript.

## Abbreviations

ZMH: Zewditu Memorial Hospital
OR: operation room
ESOPD: emergency surgical outpatient department
SRS: surgical referral clinic
LMIC: low and middle income countries
Post-op: post-operative
AAU: Addis Ababa university
CHS: College of Health Sciences
SOM: School of Medicine
HAP: hospital acquired pneumonia
